# Dietary Fiber Intake and Endometrial Cancer Risk: A Systematic Review and Meta-Analysis

**DOI:** 10.3390/nu10070945

**Published:** 2018-07-22

**Authors:** Kangning Chen, Qianyu Zhao, Xiaofan Li, Jing Zhao, Peiqin Li, Shuchun Lin, Hongwei Wang, Jiajie Zang, Ying Xiao, Wanghong Xu, Fuxue Chen, Ying Gao

**Affiliations:** 1 School of Life Sciences, Shanghai University, 333 Nanchen Road, Shanghai 200444, China; chenkangning@shu.edu.cn (K.C.); chenfuxue@staff.shu.edu.cn (F.C.); 2 CAS Key Laboratory of Nutrition, Metabolism and Food safety, Shanghai Institute of Nutrition and Health, Shanghai Institutes for Biological Sciences, University of Chinese Academy of Sciences, Chinese Academy of Sciences, 320 Yueyang Road, New Life Science Building, Room A1926, Shanghai 200031, China; zhaoqianyu2017@sibs.ac.cn (Q.Z.); xfli2018@163.com (X.L.); Zhaojing1006@126.com (J.Z.); pqlee0801@163.com (P.L.); linshuchun1992@gmail.com (S.L.); 3 DuPont Nutrition & Health, No. 10, Lane 280, Linhong Rd., Changning District, Shanghai 200335, China; hongwei.wang@dupont.com; 4 Department of Nutrition Hygiene, Division of Health Risk Factor Monitoring and Control, Shanghai Municipal Center for Disease Control and Prevention, 1380 West Zhongshan Road, Changning District, Shanghai 200336, China; zangjiajie@scdc.sh.cn; 5 Macau Institute for Applied Research in Medicine and Health, Macau University of Science and Technology, Avenide Wai Long, Taipa, Macau; yxiao@must.edu.mo; 6 Department of Epidemiology, School of Public Health, Fudan University, 138 Yi Xue Yuan Road, Shanghai 200032, China; wanghong.xu@fudan.edu.cn

**Keywords:** endometrial cancer, dietary fiber, meta-analysis, systematic review

## Abstract

Epidemiological studies are inconclusive regarding the association between dietary fiber intake and endometrial cancer risk. Thus, we aimed to conduct a meta-analysis to clarify the association between dietary fiber and endometrial cancer risk. We searched the PubMed and ISI Web databases for relevant studies through March 2018. The association between dietary fiber and endometrial cancer risk was evaluated by conducting a meta-analysis including 3 cohort and 12 case–control studies. A significant negative association was observed between total dietary fiber intake and endometrial cancer risk in 11 case–control studies (odds ratios (OR) 0.76, 95% confidence interval (CI): 0.64–0.89, *I*^2^ = 35.2%, *p* = 0.117), but a marginal positive association was observed in three cohort studies (relative risk (RR) 1.22, 95% CI: 1.00–1.49, *I*^2^ = 0.0%, *p* = 0.995). Particularly, a negative association was observed in North America (OR = 0.70, 95% CI: 0.59–0.83, *I*^2^ = 8.9%, *p* = 0.362). In addition, a positive association was observed in cereal fiber (RR = 1.26, 95% CI: 1.03–1.52, *I*^2^ = 0.0%, *p* = 0.530, 3 cohort studies) and a negative association was observed in vegetable fiber (OR = 0.74, 95% CI: 0.58–0.94, *I*^2^ = 0.0%, *p* = 0.445, 3 case–control studies). In conclusion, negative associations with endometrial cancer risk were observed for higher total dietary fiber intake and higher vegetable fiber intake in the case–control studies. However, results from the cohort studies suggested positive relationships of higher total fiber intake and higher cereal fiber intake with endometrial cancer risk.

## 1. Introduction

Endometrial cancer, ranking the fourth in incidence of all cancers in women, is the most common gynecologic cancer, especially in economically developed countries [1]. Long-lasting unopposed estrogen exposure increases the chance of development of endometrial cancer [2]. Dietary fiber is involved in absorption and reabsorption of sterols, and thus influences metabolism of estrogens. Therefore, it is biologically plausible to hypothesize an inverse association of dietary fiber intake with the risk of endometrial cancer.

Many studies have suggested that dietary fiber may decrease the risk of multiple cancers, such as colorectal cancer, breast cancer, and pancreatic cancer [3,4,5]. Several credible mechanisms have been proposed to support protective effects of dietary fiber in cancer, including decreased cholesterol levels in plasma, decreased postprandial blood sugar, and bacterial fermentation of fiber to short-chain fatty acids [6]. As a recognized healthy dietary pattern, higher dietary fiber intake reduces body fatness as reflected either by body mass index (BMI), weight, or waist circumference, hip circumference, and waist-to-hip ratio [7]. These obesity indexes were convincingly associated with an increased endometrial cancer risk [8]. High dietary fiber intake was also related with the risk of diabetes and hypertension, which are risk factors for several cancers, including endometrial cancer [9].

In 2007, Bandera et al. [10] reported an inverse association between dietary fiber intake and endometrial cancer risk by summarizing a limited number of related studies. However, the association of subtypes of dietary fiber with the risk of endometrial cancer has not been evaluated. To clarify the association, we conducted a current meta-analysis based on accumulated relevant published epidemiologic studies.

## 2. Methods

### 2.1. Search Strategy

This meta-analysis was planned, conducted, and reported according to the Preferred Reporting Items for Systematic Reviews and Meta-Analyses (PRISMA) recommendations [11] and Meta-analysis Of Observational Studies in Epidemiology (MOOSE) guidelines [12]. We systematically searched PubMed and ISI Web (up to 23 March 2018) for all studies on the associations between dietary fiber intake and endometrial cancer risk.

According to WCRF (World Cancer Research Fund) Specification Manual (available at http://www.wcrf.org), the general search terms of exposure for PubMed included diet [tiab] OR diets [tiab] OR dietetic [tiab] OR dietary [tiab] OR eating [tiab] OR intake [tiab] OR nutrient* [tiab] OR nutrition [tiab]. Specifically for dietary fiber, the key words were consistent with published in Bandera’s meta-analysis [10] including: vegetarian* [tiab] OR vegan* [tiab] OR “seventh day adventist-” [tiab] OR macrobiotic [tiab] OR food and beverages [MeSH Terms] OR fiber [tiab] OR polysaccharide* [tiab].

For the outcome terms, the key words were consistent with published in previous meta-analyses [13,14], including: (1) malign* [tiab] OR cancer* [tiab] OR carcinoma* [tiab] OR tumor* [tiab] OR tumour* [tiab]; (2) endometr* [tiab] OR corpus uteri [tiab] OR uterine [tiab]; (3) endometrial neoplasm [MeSH]; (4) #1 AND #2; (5) #3 OR #4.

### 2.2. Study Selection

**Figure 1:**
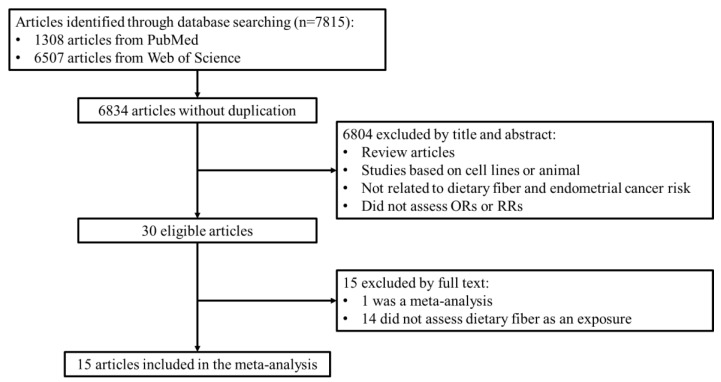
Study flow diagram.

We identified 1308 articles from PubMed and 6507 articles from the Web of Science through a database search. Subsequently, 981 duplicates were removed and 6834 articles remained. Studies were included that were: (1) case–control studies, prospective studies, or clinical trials; (2) that studied dietary fiber and other fiber subtype exposure; (3) that focused on endometrial cancer as an outcome; and (4) reported the odds ratio (OR) or relative risk between the intake of dietary fiber and the risk of endometrial cancer. Investigators identified studies based on titles, abstracts, and full texts independently. Thirty eligible articles were retrieved after titles and abstracts were reviewed. As per the exclusion criteria, (1) review articles; (2) studies based on cell lines and animals; (3) studies not related to dietary fiber and endometrial cancer risk; and (4) studies which did not assess ORs or RRs were removed. After going through the full text, 1 meta-analysis and 14 studies which did not assess dietary fiber as an exposure variable were excluded in the 30 eligible articles. Finally, 15 articles were included in this meta-analysis (Figure 1). 

### 2.3. Data Extraction

Data was extracted from each study, including last name of the first author, year of publication, country, age, sample size, number of cases, method of dietary assessment (type, time frame and number of food items), exposure (dietary fiber and other fiber subtypes), quantity of intake, hysterectomy, effect size (odds ratios (ORs) for case control study, relative risks (RRs) for cohort study), 95% confidence intervals, and adjusted variables. We presented evidence from all the selected studies, and then repeated certain analyses, excluding studies that did not meet certain quality criteria. The criteria were (1) case–control studies or cohort studies; (2) sample size of at least 200 cases for more optimal statistical power; (3) exclusion of hysterectomies from the control group; and (4) adjustment for important confounders, such as total energy and body mass index.

### 2.4. Statistical Analysis

According to the WCRF criteria (more than 2 cohort or 5 case–control studies evaluating the exposure), there were enough studies to conduct meta-analysis for total dietary fiber and cereal fiber. Although vegetable fiber, fruit fiber, and insoluble fiber did not meet the WCRF criteria, we also performed the meta-analysis for studies with vegetable fiber, and provide a table reporting the data of studies with fruit fiber and studies with insoluble fiber.

The squared inverse variance for the logarithm RR/OR was considered as the appropriate weight for each study. The Q test and *I*^2^ statistics were used to examine statistical heterogeneity among studies. As long as the *p* value for heterogeneity was less than 0.05 or the *I*^2^ statistic was more than 25%, results of the studies were considered as significantly heterogeneous and a DerSimonian and Laird random effects model was used to pool the results [15,16]. Publication bias was evaluated with funnel plots and the Egger regression asymmetry test. When the *P* value was less than 0.05, the trim and fill method was used to adjust the publication bias [17].

We used DerSimonian and Laird random effects models to calculate summary ORs, RRs, and 95% confidence intervals (CIs) for the highest versus the lowest levels of total dietary fiber, cereal fiber, and vegetable fiber. Our meta-analysis excluded the data from Shu et al. [13] because of equivocal definition of crude fiber and uncertain confidence interval. Moreover, considering the possibly analogous dietary patterns and lifestyles of North America, subgroup analyses were performed with seven studies from the USA and three studies from Canada. 

All statistical analyses were performed with STATA version 12.0 (STATA Corporation, College Station, TX, USA). All statistical tests were 2-sided.

## 3. Results

There were 3 cohort studies, 10 population-based case–control studies, and 2 hospital-based case–control studies identified for the current meta-analysis (Table 1). Overall, 14 studies reported total dietary fiber intake, 6 reported cereal fiber intake, 5 reported vegetable fiber intake, 5 reported fruit fiber intake, and 4 reported insoluble fiber intake.

### 3.1. Total Dietary Fiber

**Figure 2:**
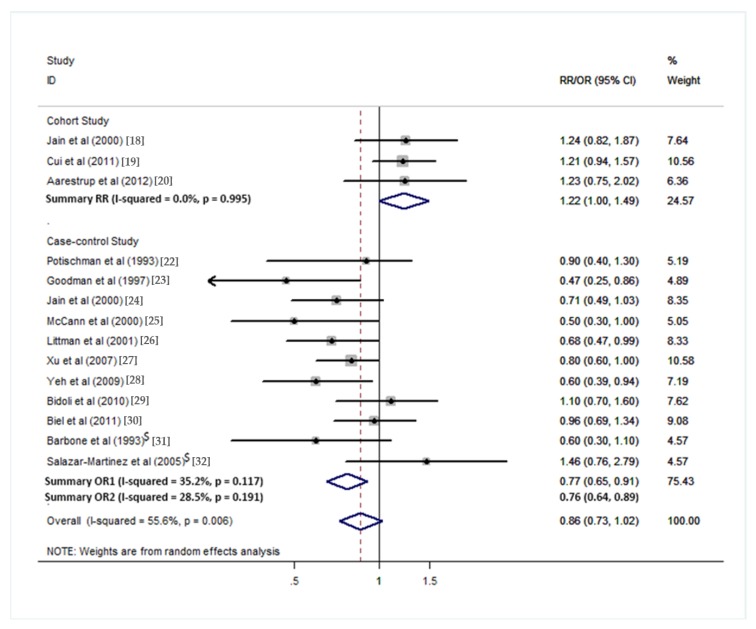
Forest plot of the summary risk estimate of endometrial cancer in the highest category of total dietary fiber intake compared with those in the lowest category. Summary OR1 is the summary risk estimate from all the case–control studies. Summary OR2 is the risk estimate from studies after exclusion. Excluded studies: ^$^Hospital-based case–control study.

**Figure 3:**
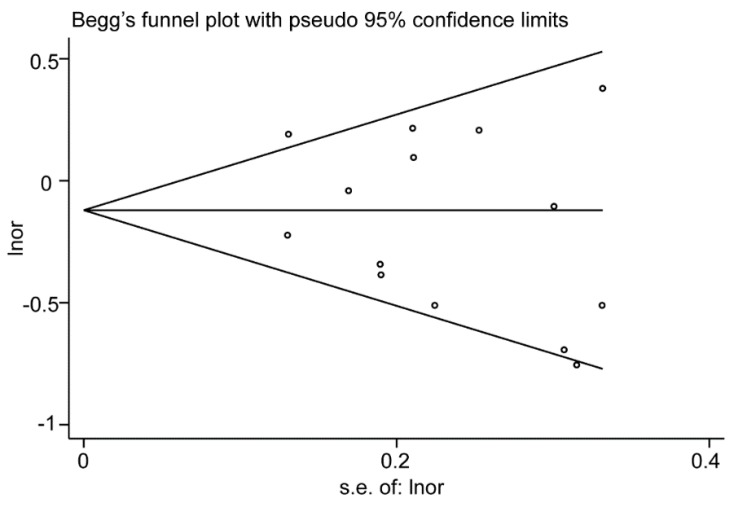
Funnel plot of the meta-analysis for the association between total dietary fiber intake and risk of endometrial cancer.

**Figure 4:**
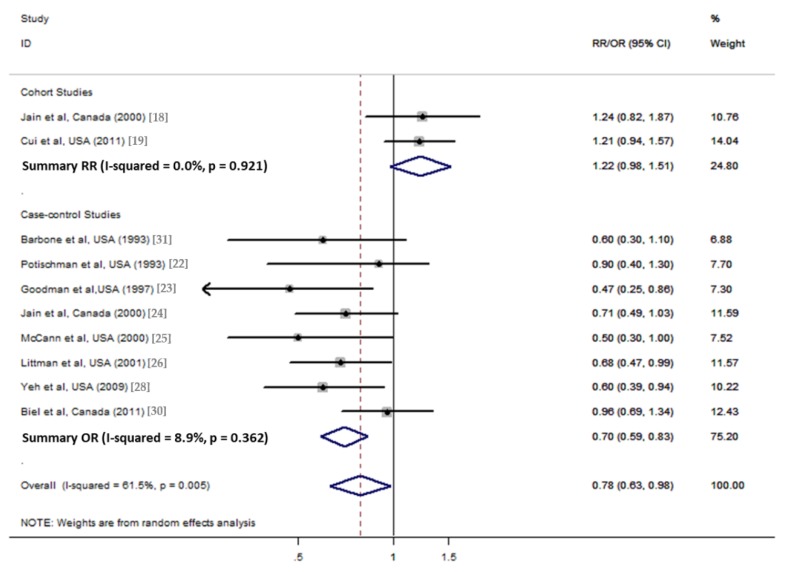
Forest plot of the summary risk estimate of endometrial cancer in the highest category of total dietary fiber intake compared with those in the lowest category of studies in North America.

Overall analysis suggested no association between total dietary fiber intake and endometrial cancer risk, which included 14 studies (3 cohorts and 11 case controls). Based on the three cohort studies, a marginal positive association was observed between dietary fiber intake and endometrial cancer risk (pooled RR = 1.22; 95% CI = 1.00–1.49; Figure 2) by comparing the highest category of dietary intake to the lowest category. For the 11 case–control studies, a negative association was observed (pooled OR1 = 0.77, 95% CI = 0.65–0.91; Figure 2). After excluding low quality studies which included less than 200 cases, the negative association was consistent (pooled OR2 = 0.76, 95% CI = 0.64–0.91; Figure 2). The Egger’s test (*p* = 0.315) and the Begg’s test (*p* = 0.324) suggested no significant publication bias (Figure 3). When stratified in North America, a significant negative association was observed (pooled OR = 0.70, 95% CI = 0.59–0.83; Figure 4).

### 3.2. Cereal Fiber Intake

**Figure 5:**
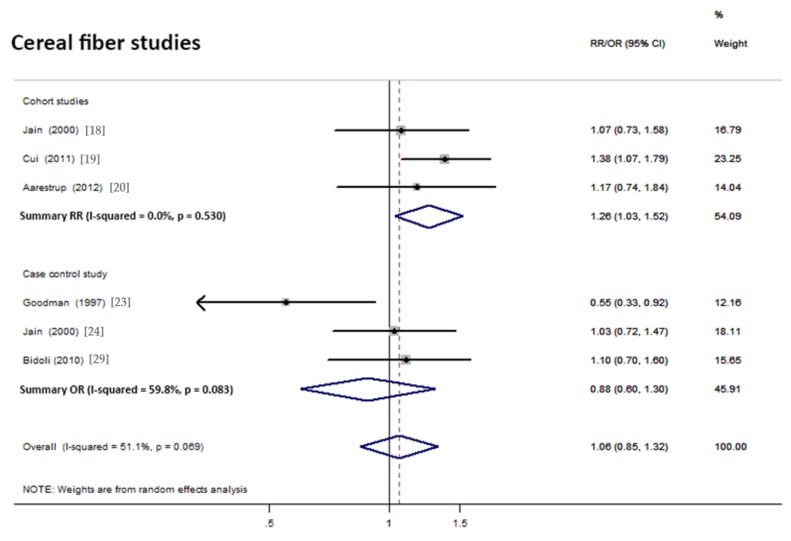
Forest plot of the summary risk estimate of endometrial cancer in the highest category of dietary cereal fiber intake compared with those in the lowest category.

Overall analysis suggested a non-significant association between cereal fiber intake and endometrial cancer risk. This analysis included 6 studies (3 cohorts and 3 case controls). Based on the three cohort studies, a significantly positive association was observed (pooled RR = 1.26; 95% CI = 1.03–1.52; Figure 5). For the three case–control studies, there was a non-significant association observed (pooled OR = 0.88, 95% CI = 0.60–1.30; Figure 5).

### 3.3. Vegetable Fiber Intake

**Figure 6:**
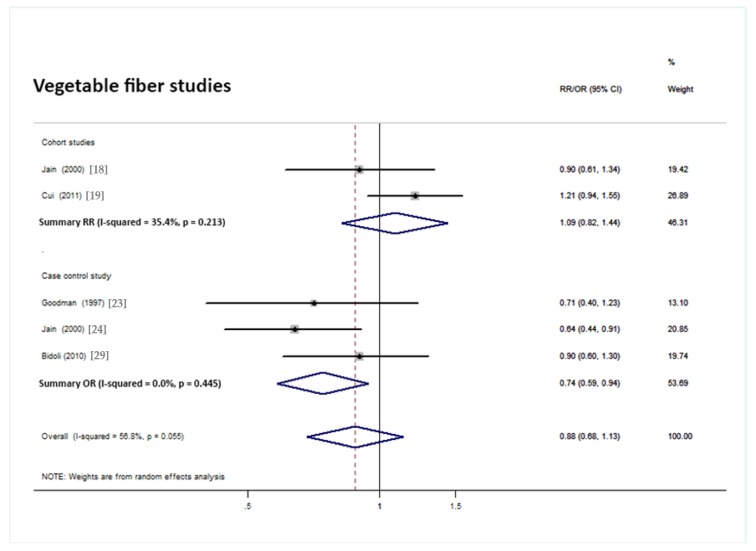
Forest plot of the summary risk estimate of endometrial cancer in the highest category of dietary vegetable fiber intake compared with those in the lowest category.

Overall analysis suggested no significant association between vegetable fiber intake and endometrial cancer risk. This included 5 studies (2 cohorts and 3 case controls). However, the summary ORs of three case–controls indicated a negative association of vegetable fiber intake with endometrial cancer risk (pooled OR = 0.74, 95% CI = 0.59 to 0.94; Figure 6).

### 3.4. Fruit Fiber Intake and Insoluble Fiber Intake

In total 5 studies (2 cohorts and 3 case controls) were included in the analysis of fruit fiber and risk of endometrial cancer. In total 4 studies (1 cohorts and 3 case controls) were included in the analysis of insoluble fiber and risk of endometrial cancer. Only Goodman et al. [23] found a negative association for fruit fiber (Table 2).

## 4. Discussion

Our meta-analysis supports an inverse association between total dietary fiber intake and risk of endometrial cancer in case-control studies, particularly in studies conducted in North America. The result of cohort studies for total fiber was inclined to a positive direction. The result of cohort studies for cereal fiber was significantly positive. Only three case–control studies support an inverse association with vegetable fiber. No significant association was observed for intake of fruit fiber and insoluble fiber. Our results for total dietary fiber derived from case–control studies is consistent with previous meta-analysis [10]. However, associations with subtypes of dietary fiber intake varied across the population. 

### 4.1. Total Dietary Fiber and Endometrial Cancer Risk

Adults who consumed large amounts of dietary fiber, compared with those who have moderate fiber intake, were at lower risk for obesity, hypertension, diabetes, cardiovascular diseases, and gastrointestinal diseases [33]. Recently, three studies respectively suggested that dietary fiber may decrease the risk of colorectal cancer [4], breast cancer [3], and pancreatic cancer [5]. The consistent inverse association for dietary fiber intake with endometrial cancer in this study strongly supports the common opinion that dietary fiber intake has health effects. 

It is a challenge to expound the biochemical mechanisms for total dietary fiber and endometrial cancer risk, for the complex components of total dietary. These analyses were based upon questionnaires in traditional epidemiologic studies, and dietary fiber was defined in different standards in questionnaires. However, substantial research has been conducted to evaluate the effect of dietary fiber and body weight, most of which showed an inverse relationship between dietary fiber intake and change in body weight [34]. WCRF analysis of global evidence shows that being overweight or obese increases the risk of 11 types of cancers, including endometrial, breast, colorectal, and pancreatic cancer [9]. Therefore, dietary fiber may influence endometrial cancer by regulating body fat.

On the other hand, bile acids (metabolites of cholesterol in liver) promote fat absorption in the intestinal, tract which is then reabsorbed to the liver by enterohepatic circulation. Dietary fiber mechanically binding bile acids results in less reabsorption of bile acids, and thus, reduces cholesterol in plasma, which is a precursor of the endogenous synthesis of estrogens. Meanwhile, absorption of fats is also restrained. Lower fat intake and lower adiposity are associated with lower endometrial cancer risk. A high-fiber diet is a healthy dietary pattern associated with lower endometrial cancer risk.

### 4.2. Subtypes of Dietary Fiber and Endometrial Cancer Risk

#### 4.2.1. Cereal Fiber

Aune et al. supported a negative association between cereal fiber and colorectal cancer risk [4]. Interestingly, our result for cereal fiber showed the opposite. In total, three cohort studies show a positive association between cereal fiber and endometrial cancer. Generally, cohort studies provide stronger evidence as compared to case–control studies in epidemiology as they are less prone to differential bias on recall of dietary habits and selection of subjects. However, whether dietary fiber intake, especially cereal fiber, reflects the intake of carbohydrate is a considerable question. Accurate assessment of cereal fiber intake and other food constituents is a challenge. Interestingly, the association between carbohydrate intake and endometrial cancer risk is positive in the three cohort studies [18,19,20]. Cereal fiber is not an independent nutrient in our diet. High cereal fiber intake could reflect high carbohydrate intake, and long-term high carbohydrate intake increases adiposity risk and endometrial cancer risk.

#### 4.2.2. Vegetable Fiber, Fruit Fiber, and Insoluble Fiber

Our meta-analysis suggested an inverse association between vegetable fiber and endometrial cancer risk, but no significant result was found for fruit fiber and insoluble fiber. Although three case–control studies support the null results, vegetable fiber and fruit fiber appeared to be protective. However here we should consider the same questions on how to accurately evaluate the biochemical constituents in these subtypes of fiber. 

### 4.3. Biochemical Constituents of Dietary Fiber

Biochemically, dietary fiber is composed of cellulose, hemicellulose, lignin, pectin, and beta-glucans. These components are resistant to digestion in small intestine and require bacterial fermentation in large intestine [6,8,35]. Lignin is partially converted to lignan in the gut. The mammalian lignans enterolactone and enterodiol are phytoestrogens which may reduce the bioavailability of steroid hormones, subsequently decreasing the risk of endometrial cancer [8,29].

Soluble fiber contains pectin, gums, inulin-type fructans, and some hemicelluloses, which have a relatively lower glycemic load. These characteristics are associated with favorable effects on glucose and insulin metabolisms and insulin resistance. Soluble fiber has also been shown to result in lower blood pressure levels and diabetes risk. Both hypertension and diabetes are risk factors for endometrial cancer [36]. Unfortunately, however, we did not have enough data to evaluate the effect of soluble fiber.

Cellulose, pectin, and beta-glucans are polysaccharides that bypasses enzymatic digestion of small intestines, but are easily degraded by microflora in colon. The gut microbiome can increase circulating hormones via deconjugation and influence the composition (or types) of hormones in circulation via hydroxylation/dehydroxylation and methylation/demethylation [37]. The composition of gut microbiome may be a factor to determine concentrations and types of circulating steroid hormones, which may increase the risk of endometrial cancer at high levels.

In addition, bacterial fermentation of fiber leads to production of short-chain fatty acids, which may have protective effects against cancer. Approximately 95% of short-chain fatty acids in the gut are absorbed and metabolized by the host for a wide range of physiological functions. Microbial-generated acetate has been shown to bind a G-protein-coupled receptor, GPR43, expressed in immune cells [38]. This suggests that the microbiota’s production of acetate may help guide the resolution of inflammatory responses. Propionic acid may also play a role in modulating T cell immune responses. Published observations reported that butyrate’s effects on cell proliferation and apoptosis appear contradictory, making it difficult to interpret its role in cancer prevention and promotion [39]. However, gut microbe is inseparably related to diet pattern and health. Discovering the roles of gut microbe in endometrial cancer should be interesting in the future.

Our meta-analysis has several strengths. This is the first time that the positive association for cereal fiber intake on endometrial cancer risk was observed by meta-analysis. Besides, we were firstly concerned with geographic effects, and observed the significantly negative association for dietary fiber intake in endometrial cancer risk in North America. It is suggested that the effects of dietary fiber intake could be related with dietary patterns and lifestyles. There are also limitations for this meta-analysis. First, there were not enough studies to explore the association between other fiber subtypes (such as soluble fiber, cellulose, and lignin) and endometrial cancer risk. Second, the results are subject to the influence of measurement error linked to the nature of food frequency questionnaire. Precise measurement of dietary fiber biochemical constituents is a challenge to understand the relationship with endometrial cancer risk. With respect to dietary fiber as a type of substrate of bacterial fermentation, it is necessary to precisely measure microflora and its metabolite to explore the relationship of dietary fiber, gut flora, and endometrial cancer risk. Finally, none of these studies analyzed different types of endometrial cancer. Type I endometrial cancer is estrogen-dependent and associated with endometrial hyperplasia, whereas type II endometrial cancer is estrogen-independent and associated with endometrial atrophy [40].

## 5. Conclusions

In conclusion, our meta-analysis observed a negative association between dietary fiber intake and endometrial cancer risk in 12 case–control studies and a significantly negative association in North America. Vegetable fiber intake tended to be negatively associated with endometrial cancer risk. Besides, the three cohort studies suggested positive associations of higher total fiber intake and higher cereal fiber intake with endometrial cancer risk. The mechanism of dietary fiber in endometrial cancer prevention warrants further research. Future prospective studies to evaluate the effect of dietary fiber should consider gut flora in relation to the risk of type I and type II tumors in endometrial cancer .

## Figures and Tables

**Table 1:** Characteristics of studies evaluating dietary fiber and endometrial cancer risk.

Reference	Country	Case-Controls (Cohort) Size	Age (Years)	Dietary Assessment	Time Frame *	Exclusion of Hysterectomy	Dietary Fiber Evaluated	OR/RR (95% CI)	Covariates Considered
**Cohort studies**									
**Jain et al. [18], 2000**	Canada	221/3697	40–59	FFQ 86 items	1 month period	Yes	Dietary fiber: >23.2 vs. <15.1 g/day Insoluble fiber Soluble fiber Cereal fiber: >4.8 vs. <2.7 g/day Fruit fiber: >5.7 vs. <2.2 g/day Vegetable fiber: >9.6 vs. <5.5 g/day	1.24 (0.82, 1.87) 1.1 (0.74, 1.63) 1.09 (0.72, 1.65) 1.07 (0.73, 1.58) 1.08 (0.73, 1.61) 0.9 (0.61, 1.34)	A.B.E.S.H.R
**Cui et al. [19], 2011**	USA	669/68070	30–55	FFQ 130 items	A 26-year period ^†^	Yes	Total fiber: 21.3 vs. 10.7 g/day (median) Vegetable fiber: 8.5 vs. 3.2 g/day (median) Fruit fiber: 7.1 vs. 1.4 g/day (median) Cereal fiber: 6 vs. 1.9 g/day (median)	1.21 (0.94, 1.57) 1.21 (0.94, 1.55) 0.97 (0.76, 1.25) 1.38 (1.07, 1.79)	B.E.S.H.R
**Aarestrup et al. [20], 2012**	Denmark	217/24418	50–64	FFQ 192 items	12 months	Yes	Total dietary fiber: >24 vs. <17 g/day Total cereal fiber: >11 vs. <7 g/day	1.23 (0.75, 2.02) 1.17 (0.74, 1.84)	B.E.S.H.R
**Population-based case–control studies**									
**Shu et al. [21], 1993**	China	268/268	18–74	FFQ 63 items	10 years	Yes	Crude fiber: >4.68 vs. <2.65 g/day	1.1	A.B.E.R
**Potischman et al. [22], 1993**	USA	399/296	20–74	FFQ 60 items (Block)	Past few years	No mention	Fiber: >13.6 vs. <7.7 g/day	0.9 (0.4, 1.3)	A.B.(E).S.H.R
**Goodman et al. [23], 1997**	USA	332/511	18–84	Dietary history 250 items	1 year	Yes	Crude fiber: >6.04 vs. <3.01 g/day NSP: >20.6 vs. <10.2 g/day Cellulose: >5.1 vs. <2.35 g/day NCP: >15 vs. <7.27 g/day Dietary fiber: >23.9 vs. <12.3 g/day Cereal fiber: >2.51 vs. <0.01 g/day Vegetable fiber: >6.25 vs. <2.94 g/day Fruit fiber: >6.27 vs. <2.21 g/day	0.6 (0.33, 1.09) 0.6 (0.32, 1.11) 0.59 (0.32, 1.07) 0.62 (0.34, 1.15) 0.47 (0.25, 0.86) 0.55 (0.33, 0.92) 0.71 (0.4, 1.23) 0.54 (0.32, 0.92)	(A).B.H.R
**Jain et al. [24], 2000**	Canada	552/562	30–79	Dietary History (unknown items)	1 year	Yes	Dietary fiber: >27.5 vs. <17.2 g/day Insoluble fiber Cereal fiber: >10.5 vs. <4.8 g/day Fruit fiber: >8.9 vs. <3.4 g/day Vegetable fiber: >12.83 vs. <6.63 g/day	0.71 (0.49, 1.03) 0.92 (0.64, 1.33) 1.03 (0.72, 1.47) 1.34 (0.92, 1.95) 0.64 (0.44, 0.91)	A.B.E.S.H.R
**McCann et al. [25], 2000**	USA	232/639	40–85	FFQ 172 items	2 years	Yes	Dietary fiber: >32 vs. <20 g/day	0.5 (0.3, 1)	A.B.E.S.H.R
**Littman et al. [26], 2001**	USA	679/944	45–74	FFQ 98 items	5 years	Yes	Total fiber: >10.7 vs.<5.6 g/1000 kcal per day	0.68 (0.47, 0.99)	A.B.E.S.H
**Xu et al. [27], 2007**	China	1204/1212	30–69	FFQ 71 items	5 years	Yes	Dietary fiber: >8.1 vs.<4.8 g/1000 kcal per day	0.8 (0.6, 1)	A.B.E.H.R
**Yeh et al. [28], 2009**	USA	541/541	27–96	FFQ 44 items	Several years	Yes	Dietary fiber: >33 vs. <16 g/day	0.6 (0.39, 0.94)	A.B.E.S.H
**Bidoli et al. [29], 2010**	Italy	454/908	18–79	FFQ 78 items	2 years	Yes	Total fiber: (mean = 15.3 ± 5.2 g/day) Soluble NCP: (mean = 7.7 ± 2.5 g/day) Total insoluble fiber: (mean = 7.6 ± 3.6 g/day) Cellulose: (mean = 3.6 ± 1.9 g/day) Insoluble NCP: (mean = 4 ± 1.5 g/day) Lignin: (mean = 1.5 ± 0.5 g/day) Vegetable fiber: (mean = 5.7 ± 2.2 g/day) Fruit fiber: (mean = 5.8 ± 3.6 g/day) Grain fiber: (mean = 4.5 ± 2.1 g/day)	1.1 (0.7, 1.6) 0.7 (0.5, 1.1) 1 (0.7, 1.5) 0.9 (0.6, 1.3) 0.9 (0.6, 1.3) 0.6 (0.4, 0.9) 0.9 (0.6, 1.3) 0.8 (0.5, 1.1) 1.1 (0.7, 1.6)	A.B.E.S.H.R
**Biel et al. [30], 2011**	Canada	506/981	30–79	Diet History Questionnaire 124 items	1 year	No mention	Dietary fiber: >21.9 vs. <14.8 g/day Insoluble fiber: >14.3 vs. <9.5 g/day Soluble fiber: >7.5 vs. <5.1 g/day	0.96 (0.69, 1.34) 0.95 (0.68, 1.34) 1.08 (0.77, 1.52)	A.B.E.H.R
**Hospital-based case-control studies**									
**Barbone et al. [31], 1993**	USA	103/236	No mention	FFQ 116 items (Willett)	1 year	No mention	Dietary fiber: 19.5 g/day	0.6 (0.3, 1.1)	A.B.E.S.H.R
**Salazar-Martinez et al. [32], 2005**	Mexico	85/629	18–81	FFQ 116 items	1 year	Yes	Dietary fiber: >24 vs. <13 g/day	1.46 (0.76, 2.79)	A.B.E.R

* Time frame for dietary assessment. ^†^ Dietary intake was assessed up to seven times over the 26-year period. FFQ, food-frequency questionnaire. NCP, non-cellulosic polysaccharides. NSP, non-starch polysaccharides; OR, odds ratio; RR, relative risk; CI, confidence interval. Covariates: A = Age, B = BMI/weight, E = total energy, S = smoking, H = hormone replacement therapy or estrogen replacement therapy, R = reproductive factors, (A): matched for age, (E): energy from carbohydrate calories.

**Table 2:** Studies that evaluated fruit fiber and insoluble fiber with respect to endometrial cancer risk.

Reference	Country	Case–Controls (Cohort) Size	Age (Year)	Type of Study	Dietary Fibers Evaluated	OR/RR (95% CI)	Covariates Considered
**Fruit fiber**							
Jain et al. [18], 2000	Canada	221/3697	40–59	Cohort study	Fruit fiber: >5.7 vs. <2.2 g/day	1.08 (0.73, 1.61)	A.B.E.S.H.R
Cui et al. [19], 2011	USA	669/68070	30–55	Cohort study	Fruit fiber: 7.1 vs. 1.4 g/day (median)	0.97 (0.76, 1.25)	B.E.S.H.R
Goodman et al. [23], 1997	USA	332/511	18–84	Population-based case-control study	Fruit fiber: >6.27 vs. <2.21 g/day	0.54 (0.32, 0.92)	(A).B.H.R
Jain et al. [24], 2000	Canada	552/562	30–79	Population-based case–control study	Fruit fiber: >8.9 vs. <3.4 g/day	1.34 (0.92, 1.95)	A.B.E.S.H.R
Bidoli et al. [29], 2010	Italy	454/908	18–79	Population-based case–control study	Fruit fiber: (mean = 5.8 ± 3.6 g/day)	0.8 (0.5, 1.1)	A.B.E.S.H.R
**Insoluble fiber**							
Jain et al. [18], 2000	Canada	221/3697	40–59	Cohort study	Insoluble fiber	1.1 (0.74, 1.63)	A.B.E.S.H.R
Jain et al. [24], 2000	Canada	552/562	30–79	Population-based case–control study	Insoluble fiber	0.92 (0.64, 1.33)	A.B.E.S.H.R
Bidoli et al. [29], 2010	Italy	454/908	18–79	Population-based case–control study	Total insoluble fiber: (mean = 7.6 ± 3.6 g/day)	1 (0.7, 1.5)	A.B.E.S.H.R
Biel et al. [30], 2011	Canada	506/981	30–79	Population-based case–control study	Insoluble fiber: >14.3 vs. <9.5 g/day	0.95 (0.68, 1.34)	A.B.E.H.R

OR, odds ratio; RR, relative risk; CI, confidence interval. Covariates: A = Age, B = BMI/weight, E = total energy, S = smoking, H = hormone replacement therapy or estrogen replacement therapy, R = reproductive factors, (A): matched for age, (E): energy from carbohydrate calories.

## References

[1] Torre A.L., Bray F., Siegel R.L., Ferlay J., Lortet-Tieulent J., Jemal A. (2015). Global Cancer Statistics, 2012. CA Cancer J. Clin..

[2] Amant F., Moerman P., Neven P., Timmerman D., van Limbergen E., Vergote I. (2005). Endometrial Cancer. Lancet.

[3] Aune D., Chan D.S.M., Greenwood D.C., Vieira A.R., Rosenblatt D.A.N., Vieira R., Norat T. (2012). Dietary Fiber and Breast Cancer Risk: A Systematic Review and Meta-Analysis of Prospective Studies. Ann. Oncol..

[4] Aune D., Chan D.S.M., Lau R., Vieira R., Greenwood D.C., Kampman E., Norat T. (2011). Dietary Fibre, Whole Grains, and Risk of Colorectal Cancer: Systematic Review and Dose-Response Meta-Analysis of Prospective Studies. Br. Med. J..

[5] Wang C.-H., Qiao C., Wang R.-C., Zhou W.-P. (2015). Dietary Fiber Intake and Pancreatic Cancer Risk: A Meta-Analysis of Epidemiologic Studies. Sci. Rep..

[6] Slavin J. (2013). Fiber and Prebiotics: Mechanisms and Health Benefits. Nutrients.

[7] Aune D., Rosenblatt D.A.N., Chan D.S.M., Vingeliene S., Abar L., Vieira A.R., Greenwood D.C., Bandera E.V., Norat T. (2015). Anthropometric Factors and Endometrial Cancer Risk: A Systematic Review and Dose-Response Meta-Analysis of Prospective Studies. Ann. Oncol..

[8] Lattimer J.M., Haub M.D. (2010). Effects of Dietary Fiber and Its Components on Metabolic Health. Nutrients.

[9] Food, Nutrition, Physical Activity, and the Prevention of Endometrial Cancer 2013.

[10] Bandera E.V., Kushi L.H., Moore D.F., Gifkins D.M., McCullough M.L. (2007). Association between Dietary Fiber and Endometrial Cancer: A Dose-Response Meta-Analysis. Am. J. Clin. Nutr..

[11] Moher D., Liberati A., Tetzlaff J., Altman D.G., Grp P. (2009). Preferred Reporting Items for Systematic Reviews and Meta-Analyses: The Prisma Statement. PLOS Med..

[12] Stroup D.F., Berlin J.A., Morton S.C., Olkin I., Williamson G.D., Rennie D., Moher D., Becker B.J., Sipe T.A., Thacker S.B. (2000). Meta-Analysis of Observational Studies in Epidemiology—A Proposal for Reporting. J. Am. Med. Assoc..

[13] Zhao J., Chen L., Gao J., Du L., Shan B., Zhang H., Wang Hu., Gao Y. (2016). Dietary Fat Intake and Endometrial Cancer Risk a Dose Response Meta-Analysis. Medicine.

[14] Bandera E.V., Kushi L.H., Moore D.F., Gifkins D.M., McCullough M.L. (2007). Consumption of Animal Foods and Endometrial Cancer Risk: A Systematic Literature Review and Meta-Analysis. Cancer Causes Control.

[15] Riley R.D., Higgins J.P.T., Deeks J.J. (2011). Interpretation of Random Effects Meta-Analyses. Br. Med. J..

[16] Dersimonian R., Laird N. (1986). Metaanalysis in Clinical-Trials. Control. Clin. Trials.

[17] Duval S., Tweedie R. (2000). Trim and Fill: A Simple Funnel-Plot-Based Method of Testing and Adjusting for Publication Bias in Meta-Analysis. Biometrics.

[18] Jain M.G., Rohan T.E., Howe G.R., Miller A.B. (2000). A Cohort Study of Nutritional Factors and Endometrial Cancer. Eur. J. Epidemiol..

[19] Cui X., Rosner B., Willett W.C., Hankinson S.E. (2011). Dietary Fat, Fiber, and Carbohydrate Intake in Relation to Risk of Endometrial Cancer. Cancer Epidemiol. Biomark. Prev..

[20] Aarestrup J., Kyro C., Christensen J., Kristensen M., Wurtz A.M.L., Johnsen N.F., Overvad K., Tjonneland A., Olsen A. (2012). Whole Grain, Dietary Fiber, and Incidence of Endometrial Cancer in a Danish Cohort Study. Nutr. Cancer Int. J..

[21] Shu X.O., Zheng W., Potischman N., Brinton L.A., Hatch M.C., Gao T., Fraumeni J.F. (1993). A Population-Based Case-Control Study of Dietary Factors and Endometrial Cancer in Shanghai, People's Republic of China. Am. J. Epidemiol..

[22] Potischman N., Swanson C.A., Brinton L.A., McAdams M., Barrett R.J., Berman M.L., Mortel R., Twiggs L.B., Wilbanks G.D., Hoover R.N. (1993). Dietary Associations in a Case-Control Study of Endometrial Cancer. Cancer Causes Control.

[23] Goodman M.T., Wilkens L.R., Hankin J.H., Lyu L.C., Wu A.H., Kolonel L.N. (1997). Association of Soy and Fiber Consumption with the Risk of Endometrial Cancer. Am. J. Epidemiol..

[24] Jain M.G., Howe G.R., Rohan T.E. (2000). Nutritional Factors and Endometrial Cancer in Ontario, Canada. Cancer Control.

[25] McCann S.E., Freudenheim J.L., Marshall J.R., Brasure J.R., Swanson M.K., Graham S. (2000). Diet in the Epidemiology of Endometrial Cancer in Western New York (United States). Cancer Causes Control.

[26] Littman A.J., Beresford S.A.A., White E. (2001). The Association of Dietary Fat and Plant Foods with Endometrial Cancer (United States). Cancer Causes Control.

[27] Xu W.-H., Dai Q., Xiang Y.B., Zhao G.M., Ruan Z.X., Cheng J.R., Zheng W., Shu X.O. (2007). Nutritional Factors in Relation to Endometrial Cancer: A Report from a Population-Based Case-Control Study in Shanghai, China. Int. J. Cancer.

[28] Yeh M., Moysich K.B., Jayaprakash V., Rodabaugh K.J., Graham S., Brasure J.R., McCann S.E. (2009). Higher Intakes of Vegetables and Vegetable-Related Nutrients Are Associated with Lower Endometrial Cancer Risks. J. Nutr..

[29] Bidoli E., Pelucchi C., Zucchetto A., Negri E., Maso L.D., Polesel J., Montella M., Franceschi S., Serraino D., la Vecchia C. (2010). Fiber Intake and Endometrial Cancer Risk. Acta Oncol..

[30] Biel R.K., Csizmadi I., Cook L.S., Courneya K.S., Magliocco A.M., Friedenreich C.M. (2011). Risk of Endometrial Cancer in Relation to Individual Nutrients from Diet and Supplements. Public Health Nutr..

[31] Barbone F., Austin H., Partridge E.E. (1993). Diet and Endometrial Cancer—A Case-Control Study. Am. J. Epidemiol..

[32] Salazar-Martinez E., Lazcano-Ponce E., Sanchez-Zamorano L.M., Gonzalez-Lira G., Escudero-DE Los Rios P., Hernandez-Avila M. (2005). Dietary Factors and Endometrial Cancer Risk. Results of a Case-Control Study in Mexico. Int. J. Gynecol. Cancer.

[33] Anderson J.W., Baird P., Davis R.H., Ferreri S., Knudtson M., Koraym A., Waters V., Williams C.L. (2009). Health Benefits of Dietary Fiber. Nutr. Rev..

[34] Dahl W.J., Stewart M.L. (2015). Position of the Academy of Nutrition and Dietetics: Health Implications of Dietary Fiber. J. Acad. Nutr. Diet..

[35] Bingham S. (1987). Definitions and Intakes of Dietary Fiber. Am. J. Clin. Nutr..

[36] Hu F.B., Manson J.E., Stampfer M.J., Colditz G., Liu S., Solomon C.G., Willett W.C. (2001). Diet, Lifestyle, and the Risk of Type 2 Diabetes Mellitus in Women. N. Engl. J. Med..

[37] Hullar M.A.J., Burnett-Hartman A.N., Lampe J.W., Zappia V., Panico S., Russo G.L., Budillon A., DellaRagione F. (2014). Gut Microbes, Diet, and Cancer. Advances in Nutrition and Cancer.

[38] Guarner F., Malagelada J.R. (2003). Gut Flora in Health and Disease. Lancet.

[39] Hague A.D., Elder J.E., Hicks D.J., Paraskeva C. (1995). Apoptosis in Colorectal Tumor-Cells - Induction by the Short-Chain Fatty-Acids Butyrate, Propionate and Acetate and by the Bile-Salt Deoxycholate. Int. J. Cancer.

[40] Murali R., Soslow R.A., Weigelt B. (2014). Classification of Endometrial Carcinoma: More Than Two Types. Lancet Oncol..

